# 单倍体造血干细胞移植治疗重型再生障碍性贫血76例疗效分析

**DOI:** 10.3760/cma.j.issn.0253-2727.2023.03.005

**Published:** 2023-03

**Authors:** 樱 张, 桂新 张, 爱明 庞, 栋林 杨, 荣莉 张, 卫华 翟, 嘉璘 魏, 祎 何, 尔烈 姜, 四洲 冯, 明哲 韩

**Affiliations:** 中国医学科学院血液病医院（中国医学科学院血液学研究所），实验血液学国家重点实验室，国家血液系统疾病临床医学研究中心，细胞生态海河实验室，天津 300020 State Key Laboratory of Experimental Hematology, National Clinical Research Center for Blood Diseases, Haihe Laboratory of Cell Ecosystem, Institute of Hematology & Blood Diseases Hospital, Chinese Academy of Medical Sciences & Peking Union Medical College, Tianjin 300020, China

**Keywords:** 再生障碍性贫血, 单倍体造血干细胞移植, 总生存期, Aplastic anemia, Haploidentical hematopoietic stem cell transplantation, Overall survival

## Abstract

**目的:**

评价单倍体造血干细胞移植（haplo-HSCT）治疗重型再生障碍性贫血（SAA）的疗效。

**方法:**

对2014年12月至2020年10月在中国医学科学院血液病医院接受haplo-HSCT的76例SAA患者进行回顾性分析。

**结果:**

①76例SAA患者中男50例，女26例，中位年龄为16（3～52）岁；SAA-Ⅰ型49例，SAA-Ⅱ型18例，肝炎相关再生障碍性贫血（HAAA）9例；外周血干细胞移植61例，骨髓移植+外周血干细胞移植15例。预处理：环磷酰胺+氟达拉滨+抗人胸腺细胞球蛋白方案46例，白消安+环磷酰胺+氟达拉滨+抗人胸腺细胞球蛋白方案30例。②3例患者移植后早期死亡而未获得粒细胞植入，8例患者未获得血小板植入。73例患者获得粒细胞植入，中位植入时间为12（9～21）d；65例患者获得血小板植入，中位植入时间为14（9～90）d。原发性植入失败发生率为10.9％，继发性植入失败的发生率为5.5％。③Ⅱ～Ⅳ度急性移植物抗宿主病（aGVHD）发生率为38.4％，Ⅲ/Ⅳ度aGVHD的发生率为16.4％，慢性移植物抗宿主病（cGVHD）的发生率为35.3％，中重度cGVHD的发生率为22.1％。④中位随访时间19.5（1～75）个月，移植后2年总生存（OS）率为（78.6±5.0）％，无失败生存（FFS）率为（75.9±5.1）％，移植相关死亡率（TRM）为（20.2±4.9）％。⑤多因素分析显示：影响haplo-HSCT后OS的危险因素包括：患者年龄>35岁（*P*＝0.008）、发生Ⅲ/Ⅳ度aGVHD（*P*＝0.008）、造血干细胞移植合并症指数（HCT-CI）评分≥3分（*P*＝0.014）、移植前铁蛋白>1500 µg/L（*P*＝0.004）、初诊中性粒细胞计数>1×10^9^/L（*P*＝0.027）。植入失败为影响患者OS及FFS的危险因素（*P*<0.001）。

**结论:**

haplo-HSCT是治疗SAA的有效方法，在儿童青少年以及年轻患者中疗效较好，发生重度aGVHD、严重感染、植入失败是影响生存率的主要因素。

## Abstract (en)

**Objective:**

The purpose of this study is to determine the efficacy of haploidentical donor hematopoietic stem cell transplantation in the treatment of severe aplastic anemia.

**Methods:**

The clinical data of 76 patients with severe aplastic anemia（SAA）patients who underwent haplo-HSCT from December 2014 to October 2020 were selectively analyzed. There were 50 males and 26 females with a median age of 16（3–52）years old. There were 49 SAA-Ⅰ patients, 18 SAA-Ⅱ patients, and 9 patients with hepatitis-associated aplastic anemia. There were 15 cases of bone marrow put together with peripheral blood stem cell transplantation and 61 cases of peripheral blood stem-cell transplantation. Conditioning regimens were Cyclophosphamide（CY）+ Fludarabine（Flu）+ATG for 46 patients and Busulfan（Bu）+ CY+Flu+ATG for 30 patients.

**Results:**

Three patients died during the myelosuppressive phase following transplantation, and 73 patients had a median time of neutrophil engraftment of 12（9–21）days; in addition to 3 patients who died early, 8 patients did not obtain platelet reconstruction after transplantation, and 65 patients had platelet engraftment with a medium time of 14（9–90）d. The incidence of primary graft failure was 10.9％ and the incidence of secondary graft failure was 5.5％. The incidence of Ⅱ–Ⅳ acute graft-versus-host disease（aGVHD）was 38.4％, the incidence of Ⅲ–Ⅳ aGVHD was 16.4％, the incidence of chronic graft anti-host disease（cGVHD）was 35.8％, and the incidence of extensive cGVHD was 22.4％. The medium follow-up time was 19.5（1–75）months, the prospective overall survival（OS）for 2 years was（78.6±5.0）％, the failure-free survival（FFS）was（75.9±5.1）％, and the transplant-related mortality was（20.2±4.9）％. Multi-factor analysis revealed that the patient older than 35 years old, Ⅲ/Ⅳ aGVHD, HCT-CI≥3, the pre-transplant ferritin ≥1 500 µg/L, the number of neutrophils >1×10^9^/L at the time of onset were risk factors affecting OS（*P*＝0.008, 0.008, 0.014, 0.004, 0.027）. Patients with graft failure had lower OS and FFS than other patients（*P*<0.001）.

**Conclusion:**

Haplo-HSCT is an effective method for treating SAA in children, adolescents, and young patients, and the occurrence of severe aGVHD and severe infection, as well as graft failure, are the main causes of survival rate. The prevention and treatment of severe aGVHD and infection are essential to improve efficacy.

再生障碍性贫血（AA）是一种由机体异常免疫应答介导的骨髓衰竭性疾病[1]。重型再生障碍性贫血（SAA）严重危及患者生命，目前主要治疗方案为免疫抑制治疗（IST）和异基因造血干细胞移植（allo-HSCT）[2]–[3]。我中心应用人类白细胞抗原（HLA）配型相合同胞供者造血干细胞移植（MSD-HSCT）治疗SAA取得了较好的疗效[4]。为评价我中心单倍体造血干细胞移植（haplo-HSCT）治疗SAA患者的疗效，本研究对2014年12月至2020年10月于我院造血干细胞移植中心接受haplo-HSCT的76例SAA患者进行回顾性分析。

## 病例与方法

一、患者资料

本研究纳入于2014年12月至2020年10月在我中心接受haplo-HSCT的76例SAA患者，参照《血液病诊断及疗效标准》[5]进行诊断。男50例，女26例，中位年龄为16（3～52）岁，移植前中位病程为4（1～237）个月。SAA-Ⅰ型49例，SAA-Ⅱ型18例，肝炎相关再生障碍性贫血（HAAA）9例。35例患者符合极重型再生障碍性贫血（VSAA）标准。SAA-Ⅱ型患者均有慢性再生障碍性贫血（CAA）病史，中位移植前病程为62.5（11～237）个月。初诊中位白细胞计数为2.64（0.14～6.8）×10^9^/L，中性粒细胞计数（ANC）为0.51（0～2.27）×10^9^/L。有19例患者伴有阵发性睡眠性血红蛋白尿（PNH）克隆，57例不伴PNH克隆。移植前71例患者接受过环孢素A（CsA）治疗，48例患者接受过雄激素类药物治疗，28例患者接受过左旋咪唑治疗，效果均欠佳。56例患者曾应用粒细胞集落刺激因子（G-CSF）治疗，其中14例有治疗反应（治疗第10天ANC升幅≥0.5×10^9^/L），52例无治疗反应。9例患者移植前抗胸腺细胞球蛋白（ATG）治疗无效（SAA-I型7例，SAA-Ⅱ型患者2例）。ATG应用至移植的中位时间为226（139～531）d。76例患者有移植前红细胞输注史，中位输注量为32（4～150）U，中位血小板输注量为304（16～1456）U，4例患者移植前曾输注富含血小板白膜，中位输注量为58（8～216）U。移植前中位血清铁蛋白为1657（150～6637）µg/L。20例患者有移植前1个月内感染史，其中8例为血流感染，病原菌包括嗜麦芽寡养单胞菌（1例）、肺炎克雷伯菌（2例）、人葡萄球菌（1例）、阴沟肠杆菌（1例）、蜡样芽孢杆菌（1例）、表皮葡萄球菌（1例）、屎肠球菌（1例），移植前血流感染均治愈；7例患者移植前发生肺部侵袭性真菌病（IFD），其中4例抗真菌治疗超过1个月无明显好转。

二、供者资料

76例患者的供者均为亲缘单倍体供者，其中HLA不全相合同胞供者21例，父母供者49例，子女供者5例，旁系亲缘供者1例。HLA高分辨配型10/10位点单倍体相合3例，1个位点不相合3例，2个位点不相合5例，3个位点不相合9例，4个位点不相合16例，5个位点不相合40例。女供男17例，男供女20例，男供男29例，女供女10例。供、患者ABO血型相合43例，主要不合9例，次要不合15例，主次均不合9例。供者中位年龄为35（8～62）岁。

三、造血干细胞来源

外周血干细胞移植61例，供者采用G-CSF 5～10 µg·kg^−1^·d^−1^进行动员，第5天采集外周血干细胞。采集后的细胞悬液进行有核细胞计数、CD34^+^细胞计数以及淋巴细胞亚群检测。采集1～3天以获得足够数量细胞。按患者体重计算采集后的细胞悬液进行有核细胞、CD34^+^细胞和CD3^+^ T细胞计数。有核细胞中位回输量为10.00（6.16～25.49）×10^8^/kg，CD34^+^细胞中位回输量为3.16（1.59～7.35）×10^6^/kg，CD3^+^T细胞中位回输量为1.73（0.52～5.15）×10^7^/kg。

骨髓联合外周血干细胞移植15例，供者采用G-CSF 5～10 µg·kg^−1^·d^−1^进行动员，动员第4天采集供者骨髓，动员第5天采集外周血干细胞，采集后的骨髓及外周血干细胞悬液分别进行有核细胞计数、CD34^+^细胞计数及淋巴细胞亚群检测。有核细胞中位回输量为8.20（6.79～14.69）×10^8^/kg，CD34^+^细胞中位回输量为4.38（3.13～8.60）×10^6^/kg，CD3^+^T细胞中位回输量为0.99（0.56～2.28）×10^7^/kg。

四、预处理方案

46例患者接受环磷酰胺（Cy）+氟达拉滨（Flu）+ATG预处理方案：Flu 30 mg·m^−2^·d^−1^×5 d，Cy总量120～160 mg/kg分3～4 d连续应用；ATG：38例应用兔抗人胸腺细胞球蛋白（rATG）2.5 mg·kg^−1^·d^−1^× 5 d，6例应用抗人T细胞猪免疫球蛋白（pATG）20 mg·kg^−1^·d^−1^×5 d，2例应用抗人T细胞兔免疫球蛋白（rATG-F）5 mg·kg^−1^·d^−1^×5 d。

30例患者接受白消安（Bu）+Cy+Flu+ATG预处理方案：Bu 3.2 mg·kg^−1^·d^−1^×2 d（或3 d）；Flu 30 mg·m^−2^·d^−1^×5 d；Cy总量80～150 mg/kg分2～4 d连续应用；ATG：12例应用rATG 2.5 mg·kg^−1^·d^−1^×5 d，17例应用pATG 20 mg·kg^−1^·d^−1^×5 d，1例应用rATG-F 5 mg·kg^−1^·d^−1^×5 d。

预处理相关不良反应依据世界卫生组织（WHO）制定的化疗急性和亚急性不良反应分级标准[6]判断，在预处理开始至移植后28 d内进行评分。

五、移植物抗宿主病（GVHD）预防

以霉酚酸酯（MMF）、短疗程甲氨蝶呤（MTX）为基础联合CsA（59例）或他克莫司（17例）预防GVHD。具体用法：预处理开始给予静脉或口服MMF 500 mg每12 h 1次至移植后1个月起逐渐减量，移植后2个月内减停；MTX 15 mg/m^2^，+1 d；MTX 10 mg/m^2^，+3 d、+6 d、+11 d静脉滴注，24 h后给予亚叶酸钙解救。预处理开始给予CsA 2 mg·kg^−1^·d^−1^或他克莫司0.03 mg·kg^−1^·d^−1^静脉滴注，移植后1个月患者胃肠道功能恢复后改为口服，移植后12～18个月逐渐减停。

六、支持治疗

所有患者均住百级层流病房，移植前应用复方磺胺甲恶唑2 g/d×7 d或预防耶氏肺孢子菌肺炎，静脉滴注更昔洛韦10 mg·kg^−1^·d^−1^×7 d或膦甲酸钠90 mg·kg^−1^·d^−1^×7 d预防巨细胞病毒（CMV）感染，移植后给予氟康唑（12例）、泊沙康唑（19例）、伊曲康唑（1例）、伏立康唑（26例）、米卡芬净（7例）、卡泊芬净（6例）预防真菌感染，1例未应用预防真菌药物，4例患者移植前合并肺IFD，预处理期间应用卡泊芬净治疗，移植后给予伏立康唑静脉滴注抗真菌治疗。

七、间充质干细胞（MSC）输注

38例患者在回输供者造血干细胞当天及第4天应用脐带来源MSC输注，输注细胞数为1×10^7^/kg。

八、疗效评价及随访

粒细胞植入：外周血ANC>0.5×10^9^/L持续3 d；血小板植入：PLT>20×10^9^/L持续7 d且脱离血小板输注。原发性植入失败：移植后28 d时中性粒细胞及血小板计数仍未达到造血重建标准。继发性植入失败：造血重建后再次出现持续的ANC<0.5×10^9^/L及PLT<20×10^9^/L伴有供者嵌合状态丢失或无复发情况下骨髓中供者细胞嵌合率<5％。移植物功能不良（PGF）：造血干细胞移植后供者完全嵌合状态下外周血细胞计数不能完全恢复（ANC≤0.5×10^9^/L，PLT≤20×10^9^/L，HGB≤70 g/L）并排除GVHD、病毒感染、药物等原因。移植后14 d、28 d及3、6、9、12、24、36、60个月进行血常规检测、骨髓细胞形态学分析及骨髓造血祖细胞培养。以脱氧核糖核酸短串联重复序列（STR）、性染色体荧光原位杂交、ABO血型检测等为检测植入的指标。随访至2021年3月1日，评价植入率、移植相关并发症的发生率、移植相关死亡率（TRM）、总生存（OS）率、无失败生存（FFS）率等指标。

九、统计学处理

采用SPSS 25.0软件进行数据分析。正态分布且方差齐的连续变量采用*t*检验进行比较，应用秩和检验对非正态分布变量进行比较。生存率的统计采用Kaplan-Meier生存曲线法。疗效相关多因素分析采用Cox回归。*P*<0.05为差异有统计学意义。

## 结果

一、预处理相关不良反应

76例患者中，22例（28.9％）患者出现恶心、呕吐、腹泻等胃肠道反应（Ⅰ度20例，Ⅱ度2例）；7例（9.2％）患者出现Ⅰ～Ⅲ度口腔溃疡（Ⅰ度5例，Ⅱ度1例，Ⅲ度1例）；30例（39.5％）患者出现转氨酶不同程度升高（Ⅰ度20例，Ⅱ度8例，Ⅲ度2例）；12例（15.8％）患者发生心功能损害（Ⅰ度9例，Ⅱ度3例）；13例（17.1％）患者出现皮疹（均为Ⅰ度皮肤损害，应用糖皮质激素治疗后均好转）。随访期间未观察到预处理相关的肺部、中枢神经系统及外周神经系统毒性。

二、造血重建

3例患者因严重感染分别于移植后第7、11、14天死亡，未获得粒细胞植入；另外73例患者获得粒细胞植入，中位植入时间为12（9～21）d；除上述3例早期死亡患者外，有8例患者移植后未获得血小板植入；65例患者获得血小板植入，中位植入时间为14（9～90）d。

三、植入失败及移植物功能不良

8例血小板未恢复患者中6例再次输注冻存供者外周血干细胞，1例血象未恢复死于脑出血合并肺部感染，1例患者血象未恢复因严重感染死亡，2例血象恢复后死于重度aGVHD，1例血象未完全恢复，需长期口服药物治疗及输血，1例血象恢复后获得长期生存，另外2例患者移植后28 d内合并严重aGVHD死亡，原发性植入失败发生率为10.9％。4例（5.5％）患者分别于移植后47、50、55、82 d发生继发性植入失败，其中3例患者输注冻存供者外周血干细胞，2例血象恢复获得长期生存，1例死于重度慢性GVHD（cGVHD）合并肺部感染，1例患者未输注供者造血干细胞，长期口服药物治疗及输血维持。6例（8.2％）患者在造血重建后发生移植物功能不良。其中2例患者口服艾曲泊帕后血象恢复，1例患者皮下注射TPO后血小板上升，1例患者输注供者分选CD34^+^外周血造血干细胞后血象恢复，2例患者回输冻存供者外周血干细胞，其中1例血小板上升，另外1例因发生cGVHD合并肺部感染死亡。

四、GVHD发生情况

除早期死亡患者外，73例患者中49例（67.1％）发生aGVHD。其中Ⅰ度aGVHD 21例，经过局部用药治疗后好转。28例（38.4％）患者发生Ⅱ～Ⅳ度aGVHD，其中12例（16.4％）为Ⅲ/Ⅳ度aGVHD。发生Ⅲ/Ⅳ度aGVHD的患者给予甲泼尼龙、CD25单抗、肿瘤坏死因子α（TNF-α）单抗、MSC、芦可替尼等治疗，5例患者因重度aGVHD合并消化道出血、肝功能衰竭、多脏器功能衰竭或严重感染死亡，7例患者病情好转。在移植后生存期>100 d的68例患者中，24例（35.8％）发生cGVHD，均表现为皮肤、口腔黏膜、眼、肝脏、肺脏受累，其中15例（22.1％）为中重度cGVHD，经甲泼尼龙、他克莫司、芦可替尼等药物治疗后4例因合并肺部感染死亡，其余11例患者病情缓解，9例（13.2％）cGVHD患者经甲泼尼龙等治疗后病情好转。

五、感染

自预处理开始至移植后，全部76例患者中22例发生血流感染，病原菌分布：肺炎克雷伯菌7例（碳青霉烯耐药3例），大肠埃希菌4例（碳青霉烯耐药1例），近平滑假丝酵母菌3例，缓症链球菌2例，铜绿假单胞菌、空肠弯曲菌、生痰二氧化碳嗜纤维菌、表皮葡萄球菌、吉拉迪嗜铜菌、鲍曼不动杆菌、嗜麦芽寡养单胞菌、嗜水气单胞菌、茄病镰刀菌各1例。发生血流感染的21例患者中3例因感染性休克、弥散性血管内凝血分别于移植后第7、11、14天死亡，病原菌分别为近平滑念珠菌、嗜麦芽寡养单胞菌合并肺炎克雷伯菌、碳青霉烯耐药肺炎克雷伯菌。其余18例患者经抗细菌或抗真菌治疗感染得到控制。29例患者移植后发生肺部感染，其中12例为肺IFD（4例为移植前肺IFD未好转，8例为新发肺IFD），经抗真菌治疗后4例好转，4例死亡（均为肺IFD）。肝脾念珠菌病、隐球菌脑膜炎各1例，均经抗真菌治疗治愈。耶氏肺孢子菌肺炎1例，以复方磺胺甲恶唑治疗后好转。44例（57.9％）患者发生CMV血症，经抗病毒治疗后血CMV-DNA转为阴性。12例（15.8％）患者发生EB病毒（EBV）血症，其中10例经抗病毒治疗后血EBV-DNA转阴，1例发生CMV肺炎，经阿昔洛韦、静脉注射免疫球蛋白治疗后好转，血EBV-DNA转阴，1例发生EBV感染相关淋巴组织增殖性疾病（B细胞型），给予利妥昔单抗375 mg/m^2^每周1次，治疗3次后病情好转，血EBV-DNA转阴。

六、死亡

至随访截止，76例患者中15例死亡，死因包括：预处理后粒细胞缺乏期血流感染3例，Ⅲ/Ⅳ度aGVHD合并消化道出血3例，合并消化道出血及肝功能衰竭1例，继发严重肺部感染2例；中重度cGVHD合并严重肺部感染4例；植入失败并发脑出血1例；移植物功能不良合并严重肺部感染1例。

七、疗效及随访

76例SAA患者中3例早期死亡，其余73例患者中位随访时间为19.5（1～75）个月。至随访截止61例存活。所有患者预期2年OS率为（78.6±5.0）％（图1），FFS为（75.9±5.1）％，TRM为（20.2±4.9）％。

**图1:**
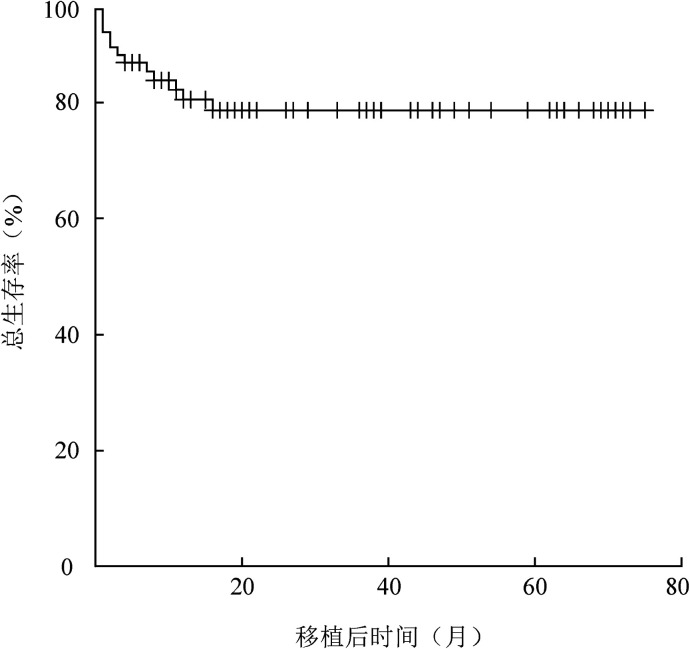
76例重型再生障碍性贫血患者单倍体造血干细胞移植后总生存曲线

八、影响疗效的相关因素分析

1. 影响OS及FFS的相关因素分析：单因素分析结果中，影响移植后OS及FFS的危险因素包括：初诊ANC>1×10^9^/L、患者移植年龄>35岁、移植前铁蛋白>1500 µg/L、移植前造血干细胞移植合并症指数（HCT-CI）评分≥3分、移植前1个月内合并感染、移植后发生Ⅲ/Ⅳ度aGVHD，移植后发生肺部感染及血流感染、诊断为非VSAA、移植后发生植入失败。而移植前红细胞输注量>20 U的患者OS率明显降低。SAA-Ⅰ患者FFS率较高。在56例移植前曾应用G-CSF治疗的患者中，有治疗反应的患者OS及FFS率明显高于无治疗反应的患者。患者性别、诊断到移植的时间、是否伴有PNH克隆、移植前是否曾应用ATG治疗、移植前血小板输注量、是否应用MSC、供者类型、HLA位点相合程度、造血干细胞来源、供者性别、供者年龄、血型相合程度、预处理是否应用Bu、预处理Cy剂量、预处理ATG种类、移植后应用CsA或他克莫司预防GVHD、有核细胞回输量、CD34^+^细胞回输量、CD3^+^T细胞回输量、是否发生Ⅰ～Ⅳ度aGVHD、是否发生中重度cGVHD、移植后是否发生CMV血症、EBV血症、是否发生移植物功能不良等因素对OS及FFS均无显著影响。详见表1。

**表1:** 影响重型再生障碍性贫血患者单倍体造血干细胞移植后生存率的单因素分析

影响因素	例数	2年OS（％）		2年FFS	
		值（％）	*P*值	值（％）	*P*值
初诊中性粒细胞计数			0.001		<0.001
≥1×10^9^/L	9	44.4±16.6		33.3±15.7	
<1×10^9^/L	67	83.4±4.9		81.9±5.0	
患者移植年龄			0.003		0.013
>35岁	9	19.4±17.2		19.4±17.2	
26~35岁	14	76.2±12.2		76.2±12.2	
≤25岁	53	86.5±4.8		82.7±5.3	
移植前铁蛋白			0.008		0.040
≤1500 µg/L	47	88.6±4.8		84.3±5.5	
>1500 µg/L	29	62.3±9.6		62.3±9.6	
移植前HCT-CI评分			<0.001		<0.001
≥3分	62	28.6±12.1		28.6±12.1	
<3分	14	91.1±3.3		87.9±4.3	
移植前1个月内感染			<0.001		<0.001
是	19	45.6±11.7		40.2±11.6	
否	57	90.9±3.9		89.2±4.2	
移植后Ⅲ/Ⅳ度aGVHD			0.004		0.016
是	12	58.3±14.2		58.3±14.2	
否	61	86.3±4.9		82.9±5.3	
移植后发生肺部感染			0.031		0.036
是	29	64.8±9.0		61.3±9.2	
否	47	89.4±4.5		87.0±5.0	
移植后发生血流感染			0.014		0.001
是	22	61.9±10.7		52.5±11.1	
否	54	85.7±5.1		85.7±5.1	
诊断为VSAA			0.035		0.012
是	35	90.9±5.0		90.9±5.0	
否	41	68.8±7.6		63.8±7.9	
移植前红细胞输注量			0.039		0.073
>20 U	50	70.2±7.2		68.1±7.4	
≤20 U	26	92.1±5.3		88.3±6.4	
移植前诊断			0.208		0.042
SAA-Ⅰ	49	81.7±5.9		81.7±5.9	
SAA-Ⅱ或HAAA	27	74.1±8.4		66.7±9.1	
植入失败			<0.001		<0.001
是	12	41.7±14.2		25.0±12.5	
否	61	90.0±4.3		90.0±4.3	
移植前G-CSF治疗反应			0.049		0.049
有	14	100		100	
无	42	70.0±7.3		70.0±7.3	
发生cGVHD			0.028		0.189
是	24	77.7±8.8		77.7±8.8	
否	43	97.7±2.3		93.0±3.9	

注 OS：总生存；FFS：无失败生存；VSAA：极重型再生障碍性贫血；SAA-Ⅰ：重型再生障碍性贫血Ⅰ型；SAA-Ⅱ：重型再生障碍性贫血Ⅱ型；HAAA：肝炎相关性再生障碍性贫血；HCT-CI：造血干细胞移植合并症指数；aGVHD：急性移植物抗宿主病；cGVHD：慢性移植物抗宿主病

多因素分析显示，患者年龄>35岁、移植前铁蛋白>1500 µg/L、HCT-CI评分≥3分、初诊中性粒细胞数>1×10^9^/L、发生Ⅲ/Ⅳ度aGVHD均为影响患者OS的危险因素，结果见表2。

**表2:** 影响重型再生障碍性贫血患者单倍体造血干细胞移植总生存的多因素分析

影响因素	*P*值	*OR*（95％ *CI*）
患者年龄>35岁	0.008	5.973（1.588~22.466）
发生Ⅲ/Ⅳ度aGVHD	0.008	24.779（2.292~267.894）
移植前1个月内发生感染	0.152	3.623（0.622~21.105）
HCT-CI评分≥3分	0.014	12.190（1.671~88.942）
移植前铁蛋白>1500 µg/L	0.004	14.242（2.381~85.173）
移植前输注红细胞	0.515	1.952（0.260~14.648）
初诊中性粒细胞计数>1×10^9^/L	0.027	0.174（0.037~0.823）
诊断为VSAA	0.258	0.369（0.066~2.076）
合并血流感染	0.274	2.748（0.449~16.796）
合并肺部感染	0.131	0.281（0.054~1.457）
发生cGVHD	0.845	0.859（0.189~3.914）

注 aGVHD：急性移植物抗宿主病；cGVHD：慢性移植物抗宿主病；HCT-CI：造血干细胞移植合并症指数；VSAA：极重型再生障碍性贫血

2. 影响GVHD发生的相关因素：在aGVHD发生方面，除早期死亡患者外，73例患者的单因素分析结果中，患者年龄、诊断至移植时间、供患者性别、供患者HLA相合程度、造血干细胞来源、移植前血清铁蛋白浓度、预处理是否包含Bu、预处理ATG类型、有核细胞回输量、CD34^+^细胞回输量、CD3^+^T细胞回输量、是否输注MSC、移植后应用CsA或他克莫司预防GVHD等因素均对Ⅱ～Ⅳ度aGVHD、Ⅲ/Ⅳ度aGVHD的发生率无影响。

在cGVHD发生方面，生存期大于100 d的68例患者的单因素分析结果显示，患者年龄、诊断至移植时间、供患者性别、供患者HLA相合程度、造血干细胞来源、预处理ATG类型、有核细胞回输量、CD34^+^细胞回输量、CD3^+^T细胞回输量、是否输注MSC、移植后应用CsA或他克莫司预防GVHD等因素对cGVHD、中重度cGVHD的发生率均无影响。在移植前血清铁蛋白浓度>1500 µg/L的患者中，重度cGVHD的发生率更高［（41.6±11.5）％对（19.4±6.7）％，*P*＝0.05］，但对于cGVHD的发生率无影响。

3. 发生植入失败的相关因素：在植入失败的发生方面，除早期死亡患者外，73例患者的单因素分析结果显示，患者诊断为非VSAA、初诊中性粒细胞数≥1×10^9^/L、供患者HLA位点5～6/10位点相合为发生植入失败的危险因素。而诊断至移植时间、造血干细胞来源、移植前血清铁蛋白浓度、移植前输注红细胞量及血小板量、供患者血型是否相合、预处理是否应用Bu、预处理应用不同剂量的Cy，预处理应用不同类型ATG、回输有核细胞数、回输CD34^+^细胞数、回输CD3^+^T细胞数、是否输注MSC等对植入失败的发生率无显著影响，结果见表3。

**表3:** 影响重型再生障碍性贫血患者单倍体造血干细胞移植植入失败发生率的单因素分析

影响因素	例数	植入失败发生率（%）	*P*值
移植前诊断			0.060
SAA-Ⅰ	48	10.4±4.0	
SAA-Ⅱ或HAAA	25	29.6±9.2	
VSAA			0.006
是	33	3.0±3.0	
否	40	28.9±7.1	
诊断至移植时间			0.699
≤12个月	58	17.2±5.0	
>12个月	15	14.3±9.4	
HLA相合程度			0.031
5~6/10位点相合	54	22.2±5.7	
7~10/10位点相合	19	0	
造血干细胞来源			0.819
骨髓+外周血	59	17.1±4.9	
外周血	14	14.3±9.4	
移植前铁蛋白			0.850
≤1 500 µg/L	47	17.0±5.5	
>1 500 µg/L	26	16.9±7.3	
初诊中性粒细胞数			0.006
≥1×10^9^/L	8	50.0±17.7	
<1×10^9^/L	65	12.4±4.1	
移植前红细胞输注量			0.428
>20 U	47	19.4±5.8	
≤20 U	26	11.5±6.3	
移植前血小板输注量			0.887
>320 U	35	17.4±6.5	
≤320 U	38	15.8±5.9	
血型相合程度			0.500
相合	42	19.0±6.1	
不相合	31	13.1±6.1	
预处理应用白消安			0.330
否	45	20.0±6.0	
是	28	10.9±5.9	
预处理环磷酰胺总量			0.175
80~120 mg/kg	10	30.0±14.5	
150~160 mg/kg	63	14.4±4.4	
预处理ATG类型			0.102
兔ATG	52	21.3±5.7	
猪ATG	21	4.8±4.6	
回输有核细胞数			0.422
≥10×10^8^/ kg	38	13.4±5.6	
<10×10^8^/ kg	35	20.0±6.8	
回输CD34^+^细胞数			0.742
<3.5×10^6^/ kg	39	15.7±5.9	
≥3.5×10^6^/ kg	34	17.6±6.5	
回输CD3^+^T细胞数			0.690
<2×10^7^/kg	46	15.2±5.3	
≥2×10^7^/kg	27	18.8±7.6	
MSC应用			
否	36	19.4±6.6	0.556
是	37	13.6±5.7	

注 SAA-Ⅰ：重型再生障碍性贫血I型；SAA-Ⅱ：重型再生障碍性贫血Ⅱ型；HAAA：肝炎相关性再生障碍性贫血；Bu：白消安；ATG：抗人胸腺细胞球蛋白；MNC：间充质干细胞

## 讨论

陈欣等[7]回顾性分析了我中心2003年1月至2012年12月接受allo-HSCT的70例SAA患者资料，结果显示，预期5年OS为（79.0±5.1）％。其中51例MSD-HSCT患者5年OS率为（85.0±5.4）％，19例替代供者移植的5年OS率为（63.2±11.1）％。近年来，随着支持治疗水平提高、ATG的使用、供者选择的优化等，全球范围内haplo-HSCT得到了广泛应用。本研究对我中心近年来接受行haplo-HSCT 的SAA患者进行了回顾性分析，虽然全部76例患者中有3例发生了早期死亡，但OS率仍达（78.6±5.0）％，尤其是在25岁及以下患者中，5年OS率为（86.5±4.8）％，与MSD-HSCT疗效相似，但>35岁患者5年OS较低。既往研究中也发现，年龄与造血干细胞移植的疗效具有很强的相关性，年龄较大的患者移植疗效较差[7]。本研究中9例35岁以上患者中5例死亡，其中4例死于感染，1例死于消化道出血。所以，SAA患者如果不具有HLA配型全相合的同胞供者，haplo-HSCT是一种替代治疗选择，适用于儿童及青少年患者。

以往研究显示，ANC较低的年轻SAA患者HSCT效果较好[8]。本研究中也发现，诊断为VSAA的患者OS及FFS明显高于其他患者，并且为SAA-Ⅰ患者的FFS率高于其他患者。初诊具有较低中性粒细胞数患者的OS率及FFS率均较高，而初诊ANC>1×10^9^/L患者的OS率仅为（44.4±16.6）％。我们认为，初诊ANC较高的部分患者具有CAA病史，研究显示，病史长、输血多的患者移植效果较差[4]，而本研究也发现移植前输注红细胞20 U以上的患者OS低于输血较少患者。既往研究显示，对于移植前病程较短且输血量较少的SAA患者，血清铁蛋白≥1 000 µg/L会明显影响患者的OS[9]。本研究中，haplo-HSCT前铁蛋白>1 500 µg/L的患者OS及FFS均较差。铁蛋白较高组患者移植前1个月感染发生率较高（33.3％对21.7％，*P*＝0.262），移植后血流感染及肺部感染等严重感染发生率也较高（60.7％对38.3％，*P*＝0.06）。因此，我们认为铁蛋白较高的患者更易发生感染，从而影响移植疗效。

细菌以及侵袭性真菌感染是导致SAA患者死亡的重要原因[10]，也是严重影响SAA患者HSCT疗效的不利因素[11]。本研究中20例患者有移植前1个月内感染史，其中4例患者移植前IFD未控制，这部分患者haplo-HSCT后OS及FFS均较差。另外，移植G-CSF治疗有效的患者行haplo-HSCT的疗效更好，可能与这些患者移植前白细胞数有所上升，发生感染较少有关。本文中3例早期死亡患者均因感染性休克死亡，其中两例合并糖尿病，可见SAA患者行haplo-HSCT前应积极防治感染控制血糖，移植后发生了30例肺部感染，22例血流感染，OS率分别仅有64.8％及61.9％，因此，移植后对于应用较强免疫抑制药物的患者以及出现了植入失败的患者，感染的监测和及时控制有利于提高疗效。

HCT-CI评分可用于预估患者进行造血干细胞移植的预后[12]，本研究中HCT-CI评分≥3分的患者OS及FFS率明显低于其他患者。因此，在移植前评估患者的各种合并症，应用HCT-CI评分评估和预测移植患者的预后，早期处理和控制各种合并症以提高移植患者的预后是在移植前需要着重考虑的问题。

MSD-HSCT研究显示，输注较多的CD34^+^细胞有利于提高OS率并降低aGVHD的发生率[13]–[14]。既往MSD-HSCT研究显示，回输CD34^+^细胞数>2.5×10^8^/kg患者aGVHD发生率明显降低[4]。本研究单因素分析显示，haplo-HSCT中输注CD34^+^细胞数的高低对患者OS及FFS均无显著影响（*P*＝0.651、0.675），aGVHD发生率也无差异（*P*＝0.355），通过分析发现，移植物有核细胞数≥10×10^8^/kg患者的OS率及FFS率均为（88.8±5.3）％，高于有核细胞数较少的患者但差异无统计学意义。我们推测增加回输物中的有核细胞数可能会有利于改善患者的生存，但仍需更多病例数进行验证。

GVHD与植入失败是移植后的常见并发症，也是影响移植疗效的重要原因。haplo-HSCT中GVHD发生率明显高于MSD-HSCT，我国研究中，Ⅱ～Ⅳ度aGVHD发生率可达30.3％～57.9％，Ⅲ～Ⅳ度aGVHD发生率为10.1％～20.1％，cGVHD发生率达到20％～40％[15]–[18]。本组病例中Ⅱ～Ⅳ度aGVHD发生率38.4％，Ⅲ/Ⅳ度aGVHD发生率为16.4％，cGVHD发生率为35.8％，显示Ⅲ/Ⅳ度aGVHD是影响移植疗效的危险因素之一，cGVHD的发生也是影响OS的危险因素。我们的患者应用Cy+Flu+ATG±Bu预处理方案，给予钙调蛋白抑制剂及短疗程MTX、MMF等预防GVHD的发生，仍有较高的aGVHD发生率，重度aGVHD发生率为16.4％，而在一项haplo-HSCT荟萃分析中显示，非清髓性预处理的患者aGVHD发生率低于包含TBI或Bu的减低强度预处理，这可能与分析中应用非清髓性预处理的研究中大多给予移植后环磷酰胺（PT-Cy）从而降低了aGVHD发生率相关，但植入失败的发生率也有所增高。我国其他中心haplo-HSCT后植入失败的发生率为6％～25％，而本研究中原发性、继发性植入失败发生率分别为10.9％、5.5％，发生植入失败的患者OS及FFS均明显低于其他患者（*P*<0.001）。在haplo-HSCT中，供者特异性抗体（DSA）是导致植入失败的关键因素[19]，而本研究中对于HLA相合位点数较多（7～10个位点相合）的患者，植入失败风险较低，可能与这部分患者中DSA阳性者较少有关。

既往MSD-HSCT研究显示，骨髓移植是SAA的首选移植方式，与外周血干细胞移植相比，具有更低的GVHD发生率[20]。国内研究中多采用骨髓+外周血干细胞移植的方式[15]。本研究中15例患者采用了骨髓+外周血干细胞移植，这组患者FFS率高于外周血干细胞移植组，重度aGVHD发生率无统计学差异，可能与本组患者例数较少有关。近年来有研究者为减少haplo-HSCT后aGVHD的发生并促进干细胞植入，在输注造血干细胞的同时予以MSC输注，发现骨髓来源MSC较脐带来源MSC具有更好的疗效[21]–[22]。也有荟萃分析发现输注MSC并不能降低aGVHD的发生[23]。本研究中输注MSC（38例）与未输注MSC的患者比较，aGVHD及植入失败的发生率差异无统计学意义。

值得注意的是，很多研究显示ATG治疗失败的患者进行挽救性haplo-HSCT疗效较差[24]–[25]。本研究中移植前曾行IST的9例患者全部存活。本研究未针对DSA对植入失败的的影响以及供患者嵌合度监测等方面进行分析，具有一定的局限性，将在今后中进一步完善数据随访以及疗效研究。

综上所述，本组病例资料显示，对于不具有HLA配型全相合同胞供者的SAA患者，haplo-HSCT可以作为一种较理想的替代治疗选择；SAA-I、初诊ANC较低的患者预后较好，而输注红细胞较多、移植前铁蛋白>1500 µg/L、移植前1个月内合并活动性感染、HCT-CI评分≥3分的患者预后较差，植入失败、重度aGVHD以及严重感染的发生是影响疗效的主要因素。
